# Work from home during COVID-19-disequilibrium of mental health and well-being among employees

**DOI:** 10.17179/excli2021-4029

**Published:** 2021-07-29

**Authors:** Nisha Shankar

**Affiliations:** 1 School of Business and Management, CHRIST University, Hosur Road, Bengaluru - 560029, Karnataka, India

## ⁯

***Dear Editor,***

The Coronavirus pandemic has disrupted the lives of many individuals globally. Employees across industries have not been spared, considering that some have been dismissed and many have been working with reduced wages. However, there are those more fortunate, with the opportunity to work from home. Regardless of the benefits of working from home, many employees have experienced heightened work stress. 

Work stress is an adverse physical or emotional reaction experienced when an employee's skills, energy, and needs do not meet the job requirements (Tongchaiprasit and Ariyabuddhiphongs, 2016[16]). Recent research has illustrated that at least half of all employees working from home are stressed out (Karatepe et al., 2018[9]). Work stress harms the employees' mental state, personal life, and behavior (Bunk and Magley, 2013[3]).

The Work from Home (WFH) concept was first coined by (Nilles,1988[13]), known at the time as "telecommuting" or "telework" (Messenger and Gschwind, 2016[12]). WFH refers to the employees' willingness to work in agile environments remote from the physical residence of the business. Currently, the WFH concept relates to employees working from home, which is made possible by the advancing technology essential for completing the job tasks remotely (Gajendran and Harrison 2007[7]; Grant et al., 2019[8]). WFH has been cited as having multiple advantages for employees, including flexibility. 

The Covid-19 pandemic accelerated the wide adoption of WFH (policies), which revealed unexplored downsides of the concept (Purwanto et al., 2020[14]; Collins and Moschler, 2009[4]). Among the key disadvantages was the negative social impact caused by reduced interactions between employees and their colleagues when working from home. Bonds created in the pre-pandemic period between colleagues were jeopardized because of the reduced social interactions (Gajendran and Harrison, 2007[7]). Employees also face the challenge of concentrating on their work due to interruptions from family members (Baruch, 2000[2]; Kazekami, 2020[10]). Thus, WFH is viewed as counteracting the advantages that led to the championing of the concept by researchers in the late 20th century.

The Covid-19 pandemic is ranked as the most stressful event in their workinglives by at least 70 % of employees. The pandemic ranks higher than stress events such as the Great Recession in 2008 and the 9/11 attack. Work stress associated with the pandemic resulted in an average loss of productivity of one hour per day for 62% of employees (Mayer, 2020[11]). The pandemic has exacerbated the stress level of employees where a survey conducted by America's State of Mind Report (Express Scripts, 2020[6]) indicates that prescriptions for medications for insomnia, anxiety, and antidepressants increased by a significance of 21 % from February 16 to March 15, 2020, which clearly illustrates the growing concerns about the health of employees as they work from home.

Work from Home (WFH) has been identified as a psychological hazard due to the increasing impact it has on work-related stress. Isolation is one of the most critical stressors during the pandemic period and directly impacts motivation. While social isolation is a typical aspect of WFH, it was more pronounced during the pandemic period as any physical contact between colleagues became impossible (Toscano and Zappalà, 2020[17]). Isolation of the employees during the social confinement imposed by governments around the world to contain the pandemic has enhanced the need to cater to the work-stress-related concerns of employees (Toscano and Zappalà, 2020[17]). The employees can face other long-term psychological issues caused by isolation, such as fear and anxiety of the pandemic and suicidal tendencies.

Another critical stressor facing the employees was maintaining a work-life balance because the boundaries between work and home were blurred. Eddleston and Mulki (2017[5]) note that WFH is associated with remote employees having difficulties disconnecting from their jobs. Thus, these employees face multiple side effects such as poor sleeping patterns and unhealthy eating habits, which creates further problems, including coronary heart diseases, diabetics, obesity, and hypertension. Moreover, the office workstation would be ergonomically compliant, which is impossible for most employees to achieve at home. Ergonomics noncompliance creates compounded issues such as musculoskeletal issues like slip disc, carpal tunnel syndrome, tennis elbow, and many other issues that can be irreversible.

Employees working from home face both mental and physical problems, which affects the employees' welfare and significantly reduces productivity. The change in the work environment and maintaining a balance between home and work creates more problems. Employees end up taking on more tasks and working for longer hours. Furthermore, there is a lack of support from management to assist the employees in managing stress, resulting in unhealthy ways to cope. This situation creates potential organizational problems such as lower employee satisfaction levels, decreased loyalty, and poor retention. This will have a huge negative impact on the productivity levels in the organization (Baptiste, 2008[1]; Sirgy, 2017[15]).

There are multiple strategies that organizations and management should adopt to improve the well-being of employees as they work from home. These strategies will be effective in the reduction of mental stress and anxiety associated with working from home.

- Management needs to be more capable of identifying and handling work stress. This can be achieved through the training of managers to enable them to quickly recognize the symptoms of work stress and implement techniques to handle them.
- Implementation of formal communication protocols can be significant for reducing work stress resulting from isolation, as managers can effectively track the state of their employees.
- The use of video conferencing improves the coordination between employees as well as allows managers and/or peers to identify employees who are having problems by observing the non-verbal cues.
- Employees can also benefit from creating no meeting zones during the day, which is crucial in achieving a work-life balance as the appropriate breaks enable them to connect more with their personal life.
- Organizations should provide employees with membership to online classes such as aerobics, yoga classes, and many others, which are responsible for breaking the sedentary lifestyle that employees occasionally fall into when working from home.
- Managers should provide resources about healthy eating and encourage employees to engage in activities that are important for their physical and mental well-being.

While remote working was popular before the pandemic, it has now become the norm. This quick adoption of WFH has highlighted the struggle of employees with achieving work-life balance, leading to work stress and increased anxiety. Managers have the responsibility of ensuring that the well-being of the employees is improved because it directly impacts the organization's productivity.

## Conflict of interest

The authors declare no conflict of interest with any commercial or other associations in connection with the submitted article.

## Financial disclosure

This research received no specific grant from any funding agency in the public, commercial, or not-for-profit sectors. 
